# 1,3-Diphenyl-3,4-dihydro­benzo[*b*][1,6]naphthyridine

**DOI:** 10.1107/S1600536810013619

**Published:** 2010-04-17

**Authors:** Werner Seebacher, Robert Weis, Robert Saf, Ferdinand Belaj

**Affiliations:** a Institute of Pharmaceutical Sciences, Departement of Pharmaceutical Chemistry, Karl-Franzens University Graz, Universitätsplatz 1, A-8010 Graz, Austria; b Institute for Chemistry and Technology of Materials (ICTM), Graz University of Technology, Stremayrgasse 16, A-8010 Graz, Austria; c Institute of Chemistry, Karl-Franzens University Graz, Schubertstrasse 1, A-8010 Graz, Austria

## Abstract

The title compound, C_24_H_18_N_2_, is the first structural example containing the 3,4-dihydro­benzo[*b*][1,6]naphthyridine fragment. It was synthesized from 2,4,6,8-tetra­phenyl-3,7-diaza­bicyclo­[3.3.1]nonan-9-one and was crystallized from a methanol–ethanol solution over two years as a racemate. The C=N double bond [1.2868 (15) Å] is bent significantly out of the plane of the aromatic bicyclic ring system [N—C—C—C = −157.63 (12)°] and out of the plane of the phenyl ring bonded at the 1-position [N—C—C—C = 41.15 (16)°].

## Related literature

For the synthesis of 1,3-diphenyl-1,2,3,4-tetra­hydro­benzo[*b*][1,6]naphthyridine, see: Sivakumar (2000 ▶). For the synthesis of 2,4,6,8-tetra­phenyl-3,7-diaza­bicyclo­[3.3.1]nonan-9-one, see Ravindran *et al.* (1991 ▶). For the crystal structures of other naphthyridine derivatives, see: Sivakumar *et al.* (2003 ▶); Laavanya *et al.* (2001 ▶).
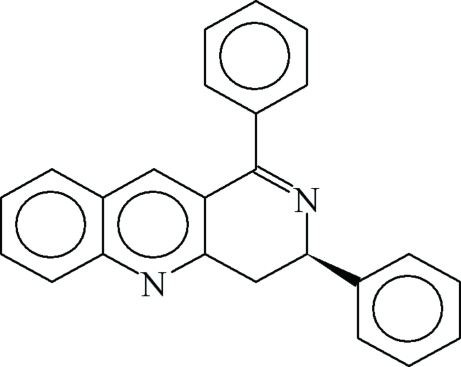


## Experimental

### 

#### Crystal data

C_24_H_18_N_2_
*M* *_r_* = 334.40
Monoclinic, 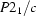

*a* = 10.2658 (4) Å
*b* = 10.8583 (5) Å
*c* = 16.1842 (7) Å
β = 107.909 (2)°
*V* = 1716.63 (13) Å^3^
*Z* = 4
Mo *K*α radiation
μ = 0.08 mm^−1^
*T* = 100 K
0.32 × 0.28 × 0.16 mm

#### Data collection

Bruker APEXII CCD diffractometer
Absorption correction: multi-scan (*SADABS*; Bruker, 2000 ▶) *T*_min_ = 0.885, *T*_max_ = 0.988
20360 measured reflections
3365 independent reflections
3060 reflections with *I* > 2σ(*I*)
*R*_int_ = 0.029

#### Refinement

*R*[*F* ^2^ > 2σ(*F* ^2^)] = 0.036
*wR*(*F* ^2^) = 0.094
*S* = 1.02
3365 reflections
241 parameters
Only H-atom displacement parameters refined
Δρ_max_ = 0.40 e Å^−3^
Δρ_min_ = −0.28 e Å^−3^

### 

Data collection: *APEX2* (Bruker, 2004 ▶); cell refinement: *SAINT* (Bruker, 2000 ▶); data reduction: *SAINT*; program(s) used to solve structure: *SHELXS97* (Sheldrick, 2008 ▶); program(s) used to refine structure: *SHELXL97* (Sheldrick, 2008 ▶); molecular graphics: modified *ORTEP* (Johnson, 1965 ▶); software used to prepare material for publication: *SHELXL97*.

## Supplementary Material

Crystal structure: contains datablocks global, I. DOI: 10.1107/S1600536810013619/bt5246sup1.cif

Structure factors: contains datablocks I. DOI: 10.1107/S1600536810013619/bt5246Isup2.hkl

Additional supplementary materials:  crystallographic information; 3D view; checkCIF report

## Figures and Tables

**Fig. 1.**
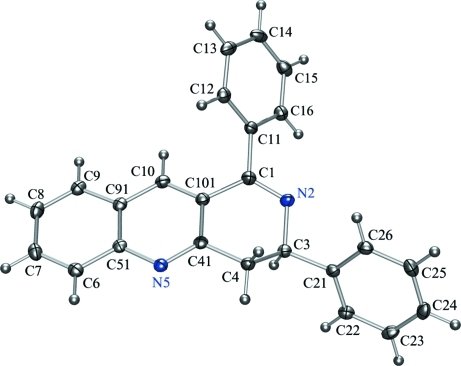
ORTEP plot (Johnson, 1965) showing the atomic numbering scheme. The probability ellipsoids are drawn at the 50% probability level, the H atoms are drawn with arbitrary radii. Selected distances: C1—N2 1.2868 (15) Å, C1—C101 1.4862 (16) Å, C1—C11 1.4936 (16) Å, N2—C3 1.4769 (14) Å, C3—C21 1.5128 (15) Å, C3—C4 1.5369 (15) Å, C4—C41 1.5073 (15) Å, C41—N5 1.3152 (14) Å, C41—C101 1.4293 (15) Å, N5—C51 1.3764 (14) Å.

## supplementary crystallographic information

### Comment

Schmidt reaction of 2,4,6,8-tetraphenyl-3,7-diazabicyclo[3.3.1]nonan-9-one leads not to the expected bicyclic amide but to 1,2,3,4-tetrahydrobenzo[*b*][1,6]naphthyridine. This compound crystallized in form of fine needles from ethanol. Our attempts to yield more compact crystals ended up in a new compound, 1,3-diphenyl-3,4-dihydrobenzo[*b*][1,6]naphthyridine, which was formed after a lomg term crystallization from a mixture of ethanol and methanol.

### Experimental

Synthesis: 2,4,6,8-Tetraphenyl-3,7-diazabicyclo[3.3.1]nonan-9-one (1.34 g, 3.0 mmol) was dissolved in concentrated sulfuric acid (4 ml) under stirring and cooling and by use of an ultrasonic bath. This takes some time and the solution comes up to room temperature during this process. When the substance is completely dissolved, NaN_3_ (240 mg, 3,7 mmol) was added and the reaction mixture was stirred for 1 h at room temperature. It was quenched with ice water, the yellow solution was extracted 3 times with ether to remove non basic impurities and then alkalized with 2 M NaOH solution. Then it was extracted 5 times with CH_2_Cl_2_, the organic layers were combined, washed 3 times with water, dried (Na_2_SO_4_), filtered and the solvent removed in vacuo. The residue was dissolved in benzene and filtered and the solvent removed in vacuo. Finally, the residue was dissolved in the minimum amount of hot ethanol and the solution left for crystallization for 2 days. The formed needles were sucked off and dried giving pure 1,3-diphenyl-1,2,3,4-tetrahydro-benzo[b][1,6]naphthyridine (330 mg, 0.98 mmol, 33 % yield). A part of it was dissolved in a mixture of methanol and ethanol and left for crystallization for 2 years. A few crystals of 1,3-diphenyl-3,4-dihydro-benzo[b][1,6]naphthyridine were obtained and subjected to the x-ray structure analysis.

HR—MS data [collected on a GCT-Premier spectrometer, Waters (EI, 70 eV)]: C_24_H_18_N_2_ requires [M]^+^ 334.1470; Found: 334.1452; C_24_H_17_N_2_ requires [M—H]^+^ 333.1392; Found: 333.1381.

### Crystal data

**Table:**

C_24_H_18_N_2_	*F*(000) = 704
*M**_r_* = 334.40	*D*_x_ = 1.294 Mg m^−^^3^
Monoclinic, *P*2_1_/*c*	Mo *K*α radiation, λ = 0.71073 Å
Hall symbol: -P 2ybc	Cell parameters from 9913 reflections
*a* = 10.2658 (4) Å	θ = 2.7–26.0°
*b* = 10.8583 (5) Å	µ = 0.08 mm^−^^1^
*c* = 16.1842 (7) Å	*T* = 100 K
β = 107.909 (2)°	Block, yellow
*V* = 1716.63 (13) Å^3^	0.32 × 0.28 × 0.16 mm
*Z* = 4	

### Data collection

**Table:**

Bruker APEXII CCD diffractometer	3365 independent reflections
Radiation source: sealed tube	3060 reflections with *I* > 2σ(*I*)
graphite	*R*_int_ = 0.029
φ and ω scans	θ_max_ = 26.0°, θ_min_ = 2.3°
Absorption correction: multi-scan (*SADABS*; Bruker, 2000)	*h* = −12→12
*T*_min_ = 0.885, *T*_max_ = 0.988	*k* = −13→13
20360 measured reflections	*l* = −19→19

### Refinement

**Table:**

Refinement on *F*^2^	Primary atom site location: structure-invariant direct methods
Least-squares matrix: full	Secondary atom site location: difference Fourier map
*R*[*F*^2^ > 2σ(*F*^2^)] = 0.036	Hydrogen site location: inferred from neighbouring sites
*wR*(*F*^2^) = 0.094	Only H-atom displacement parameters refined
*S* = 1.02	*w* = 1/[σ^2^(*F*_o_^2^) + (0.0446*P*)^2^ + 0.7644*P*] where *P* = (*F*_o_^2^ + 2*F*_c_^2^)/3
3365 reflections	(Δ/σ)_max_ < 0.001
241 parameters	Δρ_max_ = 0.40 e Å^−^^3^
0 restraints	Δρ_min_ = −0.28 e Å^−^^3^

### Special details

**Table:**

Geometry. All esds (except the esd in the dihedral angle between two l.s. planes) are estimated using the full covariance matrix. The cell esds are taken into account individually in the estimation of esds in distances, angles and torsion angles; correlations between esds in cell parameters are only used when they are defined by crystal symmetry. An approximate (isotropic) treatment of cell esds is used for estimating esds involving l.s. planes.
Refinement. Refinement of *F*^2^ against ALL reflections. The weighted *R*-factor wR and goodness of fit *S* are based on *F*^2^, conventional *R*-factors *R* are based on *F*, with *F* set to zero for negative *F*^2^. The threshold expression of *F*^2^ > σ(*F*^2^) is used only for calculating *R*-factors(gt) etc. and is not relevant to the choice of reflections for refinement. *R*-factors based on *F*^2^ are statistically about twice as large as those based on *F*, and *R*- factors based on ALL data will be even larger.The non-hydrogen atoms were refined with anisotropic displacement parameters without any constraints. The H atom of the tertiary C—H group was refined with an individual isotropic displacement parameter and all X—C—H angles equal at a C—H distance of 1.00 Å (AFIX 13 of SHELXL-97). The H atoms of the CH_2_ group were refined with common isotropic displacement parameters and idealized geometry with approximately tetrahedral angles and C—H distances of 0.99 Å (AFIX 23 of SHELXL-97). The H atoms of the phenyl rings as well as the atoma H6, H7, H8, and H9 were put at the external bisector of the C—C—C angle at a C—H distance of 0.95 Å and common isotropic displacement parameters were refined for the H atoms of the same ring (AFIX 43 of SHELXL-97). The H atom H10 was put at the external bisector of the C—C—C angle at a C—H distance of 0.95 Å but the individual isotropic displacement parameter was free to refine (AFIX 43 of SHELXL-97).

### Fractional atomic coordinates and isotropic or equivalent isotropic displacement parameters (Å^2^)

**Table:**

	*x*	*y*	*z*	*U*_iso_*/*U*_eq_	
C1	0.60895 (12)	0.61918 (11)	0.71264 (8)	0.0221 (3)	
N2	0.67352 (10)	0.72215 (9)	0.71977 (6)	0.0183 (2)	
C3	0.61969 (11)	0.81335 (10)	0.64987 (7)	0.0162 (2)	
H3	0.5364	0.8516	0.6588	0.016 (3)*	
C4	0.57620 (11)	0.75231 (10)	0.55983 (7)	0.0167 (2)	
H41	0.5341	0.8145	0.5148	0.022 (2)*	
H42	0.6576	0.7180	0.5474	0.022 (2)*	
C41	0.47489 (11)	0.65074 (10)	0.55714 (7)	0.0154 (2)	
N5	0.37687 (9)	0.62770 (8)	0.48450 (6)	0.0160 (2)	
C51	0.28123 (11)	0.53968 (10)	0.48615 (7)	0.0160 (2)	
C6	0.17392 (11)	0.51425 (11)	0.40906 (7)	0.0186 (2)	
H6	0.1692	0.5578	0.3573	0.0233 (17)*	
C7	0.07669 (12)	0.42730 (11)	0.40843 (8)	0.0216 (3)	
H7	0.0054	0.4109	0.3561	0.0233 (17)*	
C8	0.08147 (12)	0.36181 (11)	0.48477 (8)	0.0220 (3)	
H8	0.0143	0.3010	0.4833	0.0233 (17)*	
C9	0.18252 (12)	0.38568 (11)	0.56076 (8)	0.0195 (2)	
H9	0.1839	0.3429	0.6122	0.0233 (17)*	
C91	0.28508 (11)	0.47405 (10)	0.56299 (7)	0.0168 (2)	
C10	0.39474 (12)	0.49926 (10)	0.63871 (7)	0.0180 (2)	
H10	0.4019	0.4564	0.6911	0.022 (3)*	
C101	0.49068 (11)	0.58561 (10)	0.63637 (7)	0.0173 (2)	
C11	0.66051 (11)	0.52633 (10)	0.78325 (7)	0.0173 (2)	
C12	0.67192 (11)	0.40231 (11)	0.76360 (7)	0.0182 (2)	
H12	0.6417	0.3754	0.7049	0.0262 (16)*	
C13	0.72750 (11)	0.31812 (11)	0.82995 (8)	0.0217 (3)	
H13	0.7371	0.2341	0.8164	0.0262 (16)*	
C14	0.76892 (12)	0.35674 (12)	0.91590 (8)	0.0234 (3)	
H14	0.8049	0.2988	0.9611	0.0262 (16)*	
C15	0.75784 (12)	0.47979 (12)	0.93583 (8)	0.0236 (3)	
H15	0.7867	0.5062	0.9946	0.0262 (16)*	
C16	0.70445 (12)	0.56423 (11)	0.86972 (8)	0.0208 (3)	
H16	0.6978	0.6486	0.8835	0.0262 (16)*	
C21	0.72353 (11)	0.91467 (10)	0.65640 (7)	0.0154 (2)	
C22	0.68122 (12)	1.03683 (11)	0.64243 (7)	0.0184 (2)	
H22	0.5871	1.0563	0.6307	0.0257 (16)*	
C23	0.77463 (13)	1.13070 (11)	0.64538 (7)	0.0210 (3)	
H23	0.7441	1.2136	0.6357	0.0257 (16)*	
C24	0.91240 (12)	1.10332 (11)	0.66241 (7)	0.0218 (3)	
H24	0.9766	1.1672	0.6646	0.0257 (16)*	
C25	0.95581 (12)	0.98180 (12)	0.67630 (8)	0.0220 (3)	
H25	1.0500	0.9626	0.6876	0.0257 (16)*	
C26	0.86247 (12)	0.88817 (11)	0.67380 (7)	0.0190 (2)	
H26	0.8934	0.8055	0.6840	0.0257 (16)*	

### Atomic displacement parameters (Å^2^)

**Table:**

	*U*^11^	*U*^22^	*U*^33^	*U*^12^	*U*^13^	*U*^23^
C1	0.0239 (6)	0.0193 (6)	0.0199 (6)	−0.0017 (5)	0.0022 (5)	0.0022 (5)
N2	0.0227 (5)	0.0168 (5)	0.0146 (5)	−0.0015 (4)	0.0046 (4)	0.0017 (4)
C3	0.0175 (5)	0.0155 (5)	0.0157 (5)	0.0009 (4)	0.0051 (4)	0.0005 (4)
C4	0.0197 (5)	0.0160 (5)	0.0139 (5)	−0.0009 (4)	0.0043 (4)	0.0014 (4)
C41	0.0166 (5)	0.0135 (5)	0.0165 (5)	0.0029 (4)	0.0059 (4)	−0.0005 (4)
N5	0.0173 (5)	0.0144 (5)	0.0161 (5)	0.0017 (4)	0.0047 (4)	−0.0008 (4)
C51	0.0170 (5)	0.0133 (5)	0.0182 (5)	0.0029 (4)	0.0061 (4)	−0.0016 (4)
C6	0.0196 (6)	0.0181 (6)	0.0176 (6)	0.0033 (4)	0.0050 (4)	−0.0012 (4)
C7	0.0176 (6)	0.0225 (6)	0.0226 (6)	0.0008 (5)	0.0031 (5)	−0.0061 (5)
C8	0.0181 (6)	0.0198 (6)	0.0293 (6)	−0.0038 (4)	0.0091 (5)	−0.0038 (5)
C9	0.0205 (6)	0.0173 (6)	0.0227 (6)	0.0005 (4)	0.0098 (5)	−0.0007 (4)
C91	0.0178 (5)	0.0140 (5)	0.0194 (6)	0.0022 (4)	0.0071 (4)	−0.0017 (4)
C10	0.0218 (6)	0.0159 (5)	0.0169 (5)	0.0014 (4)	0.0067 (5)	0.0015 (4)
C101	0.0191 (6)	0.0150 (5)	0.0169 (6)	0.0014 (4)	0.0043 (4)	0.0003 (4)
C11	0.0151 (5)	0.0186 (6)	0.0177 (5)	−0.0011 (4)	0.0042 (4)	0.0029 (4)
C12	0.0164 (5)	0.0204 (6)	0.0192 (6)	−0.0019 (4)	0.0074 (4)	0.0001 (4)
C13	0.0171 (5)	0.0176 (6)	0.0328 (7)	0.0015 (4)	0.0112 (5)	0.0048 (5)
C14	0.0165 (6)	0.0288 (7)	0.0252 (6)	0.0029 (5)	0.0067 (5)	0.0138 (5)
C15	0.0206 (6)	0.0339 (7)	0.0158 (6)	−0.0013 (5)	0.0046 (5)	0.0035 (5)
C16	0.0204 (6)	0.0211 (6)	0.0197 (6)	−0.0004 (5)	0.0043 (5)	−0.0008 (5)
C21	0.0191 (5)	0.0164 (5)	0.0104 (5)	−0.0003 (4)	0.0040 (4)	−0.0005 (4)
C22	0.0199 (6)	0.0190 (6)	0.0172 (5)	0.0031 (4)	0.0068 (4)	0.0012 (4)
C23	0.0318 (6)	0.0141 (5)	0.0180 (6)	0.0006 (5)	0.0091 (5)	−0.0006 (4)
C24	0.0265 (6)	0.0221 (6)	0.0157 (5)	−0.0096 (5)	0.0051 (5)	−0.0034 (5)
C25	0.0169 (6)	0.0287 (6)	0.0183 (6)	−0.0010 (5)	0.0024 (4)	−0.0013 (5)
C26	0.0207 (6)	0.0168 (6)	0.0177 (5)	0.0031 (4)	0.0033 (4)	0.0004 (4)

### Geometric parameters (Å, °)

**Table:**

C1—N2	1.2868 (15)	C10—C101	1.3688 (16)
C1—C101	1.4862 (16)	C10—H10	0.95
C1—C11	1.4936 (16)	C11—C16	1.3938 (16)
N2—C3	1.4769 (14)	C11—C12	1.3969 (16)
C3—C21	1.5128 (15)	C12—C13	1.3920 (16)
C3—C4	1.5369 (15)	C12—H12	0.95
C3—H3	1.00	C13—C14	1.3884 (18)
C4—C41	1.5073 (15)	C13—H13	0.95
C4—H41	0.99	C14—C15	1.3873 (19)
C4—H42	0.99	C14—H14	0.95
C41—N5	1.3152 (14)	C15—C16	1.3875 (17)
C41—C101	1.4293 (15)	C15—H15	0.95
N5—C51	1.3764 (14)	C16—H16	0.95
C51—C6	1.4146 (16)	C21—C22	1.3925 (16)
C51—C91	1.4233 (16)	C21—C26	1.3962 (16)
C6—C7	1.3717 (17)	C22—C23	1.3901 (17)
C6—H6	0.95	C22—H22	0.95
C7—C8	1.4132 (17)	C23—C24	1.3874 (17)
C7—H7	0.95	C23—H23	0.95
C8—C9	1.3679 (17)	C24—C25	1.3887 (18)
C8—H8	0.95	C24—H24	0.95
C9—C91	1.4168 (16)	C25—C26	1.3891 (17)
C9—H9	0.95	C25—H25	0.95
C91—C10	1.4126 (16)	C26—H26	0.95
			
N2—C1—C101	123.67 (11)	C91—C10—H10	120.1
N2—C1—C11	117.79 (10)	C10—C101—C41	118.67 (10)
C101—C1—C11	118.47 (10)	C10—C101—C1	123.78 (10)
C1—N2—C3	116.92 (9)	C41—C101—C1	117.52 (10)
N2—C3—C21	110.15 (9)	C16—C11—C12	119.29 (10)
N2—C3—C4	111.61 (9)	C16—C11—C1	119.87 (11)
C21—C3—C4	111.89 (9)	C12—C11—C1	120.75 (10)
N2—C3—H3	107.7	C13—C12—C11	120.04 (11)
C21—C3—H3	107.7	C13—C12—H12	120.0
C4—C3—H3	107.7	C11—C12—H12	120.0
C41—C4—C3	109.85 (9)	C14—C13—C12	120.07 (11)
C41—C4—H41	109.7	C14—C13—H13	120.0
C3—C4—H41	109.7	C12—C13—H13	120.0
C41—C4—H42	109.7	C15—C14—C13	120.13 (11)
C3—C4—H42	109.7	C15—C14—H14	119.9
H41—C4—H42	108.2	C13—C14—H14	119.9
N5—C41—C101	123.53 (10)	C14—C15—C16	119.89 (11)
N5—C41—C4	119.76 (10)	C14—C15—H15	120.1
C101—C41—C4	116.69 (9)	C16—C15—H15	120.1
C41—N5—C51	117.96 (9)	C15—C16—C11	120.55 (11)
N5—C51—C6	119.02 (10)	C15—C16—H16	119.7
N5—C51—C91	122.44 (10)	C11—C16—H16	119.7
C6—C51—C91	118.54 (10)	C22—C21—C26	118.52 (10)
C7—C6—C51	120.56 (11)	C22—C21—C3	120.23 (10)
C7—C6—H6	119.7	C26—C21—C3	121.23 (10)
C51—C6—H6	119.7	C23—C22—C21	121.02 (11)
C6—C7—C8	120.68 (11)	C23—C22—H22	119.5
C6—C7—H7	119.7	C21—C22—H22	119.5
C8—C7—H7	119.7	C24—C23—C22	120.00 (11)
C9—C8—C7	120.24 (11)	C24—C23—H23	120.0
C9—C8—H8	119.9	C22—C23—H23	120.0
C7—C8—H8	119.9	C23—C24—C25	119.51 (11)
C8—C9—C91	120.22 (11)	C23—C24—H24	120.2
C8—C9—H9	119.9	C25—C24—H24	120.2
C91—C9—H9	119.9	C24—C25—C26	120.43 (11)
C10—C91—C9	122.76 (11)	C24—C25—H25	119.8
C10—C91—C51	117.49 (10)	C26—C25—H25	119.8
C9—C91—C51	119.74 (10)	C25—C26—C21	120.52 (11)
C101—C10—C91	119.80 (10)	C25—C26—H26	119.7
C101—C10—H10	120.1	C21—C26—H26	119.7
			
C101—C1—N2—C3	3.86 (17)	C4—C41—C101—C1	−3.06 (15)
C11—C1—N2—C3	−179.16 (10)	N2—C1—C101—C10	−157.63 (12)
C1—N2—C3—C21	−166.72 (10)	C11—C1—C101—C10	25.41 (17)
C1—N2—C3—C4	−41.79 (14)	N2—C1—C101—C41	20.27 (17)
N2—C3—C4—C41	55.03 (12)	C11—C1—C101—C41	−156.69 (10)
C21—C3—C4—C41	178.98 (9)	N2—C1—C11—C16	41.15 (16)
C3—C4—C41—N5	146.44 (10)	C101—C1—C11—C16	−141.71 (11)
C3—C4—C41—C101	−32.39 (13)	N2—C1—C11—C12	−135.55 (12)
C101—C41—N5—C51	2.88 (16)	C101—C1—C11—C12	41.59 (16)
C4—C41—N5—C51	−175.87 (9)	C16—C11—C12—C13	−0.41 (16)
C41—N5—C51—C6	179.30 (10)	C1—C11—C12—C13	176.31 (10)
C41—N5—C51—C91	0.11 (15)	C11—C12—C13—C14	1.41 (17)
N5—C51—C6—C7	−179.86 (10)	C12—C13—C14—C15	−1.41 (17)
C91—C51—C6—C7	−0.64 (16)	C13—C14—C15—C16	0.39 (18)
C51—C6—C7—C8	0.24 (17)	C14—C15—C16—C11	0.61 (18)
C6—C7—C8—C9	0.89 (18)	C12—C11—C16—C15	−0.60 (17)
C7—C8—C9—C91	−1.58 (17)	C1—C11—C16—C15	−177.35 (11)
C8—C9—C91—C10	−177.57 (11)	N2—C3—C21—C22	−139.22 (10)
C8—C9—C91—C51	1.16 (17)	C4—C3—C21—C22	96.01 (12)
N5—C51—C91—C10	−2.06 (16)	N2—C3—C21—C26	42.74 (13)
C6—C51—C91—C10	178.75 (10)	C4—C3—C21—C26	−82.02 (12)
N5—C51—C91—C9	179.14 (10)	C26—C21—C22—C23	0.20 (16)
C6—C51—C91—C9	−0.05 (16)	C3—C21—C22—C23	−177.89 (10)
C9—C91—C10—C101	179.82 (10)	C21—C22—C23—C24	0.03 (17)
C51—C91—C10—C101	1.06 (16)	C22—C23—C24—C25	0.09 (17)
C91—C10—C101—C41	1.65 (16)	C23—C24—C25—C26	−0.43 (17)
C91—C10—C101—C1	179.53 (10)	C24—C25—C26—C21	0.66 (17)
N5—C41—C101—C10	−3.83 (17)	C22—C21—C26—C25	−0.54 (16)
C4—C41—C101—C10	174.95 (10)	C3—C21—C26—C25	177.53 (10)
N5—C41—C101—C1	178.16 (10)		
